# Corneal hysteresis as a risk factor for optic nerve head surface depression and retinal nerve fiber layer thinning in glaucoma patients

**DOI:** 10.1038/s41598-021-90588-7

**Published:** 2021-06-03

**Authors:** Guihua Xu, Zilin Chen

**Affiliations:** Department of Ophthalmology, Huizhou Municipal Central Hospital, No.41, Eling North Road, Huizhou, 516001 China

**Keywords:** Risk factors, Optic nerve diseases

## Abstract

To evaluate the role of corneal hysteresis (CH) as a risk factor for progressive ONH surface depression and RNFL thinning measured by confocal scanning laser ophthalmoscopy (CSLO) and spectral-domain optical coherence tomography (SD-OCT), respectively in glaucoma patients. Prospective study. A total of 146 eyes of 90 patients with glaucoma were recruited consecutively. The CH measurements were acquired at baseline and 4-months interval using the Ocular Response Analyzer (Reichert Instruments, Depew, NY). Eyes were imaged by CSLO (Heidelberg Retinal Tomograph [HRT]; Heidelberg Engineering, GmbH, Dossenheim, Germany) and SD-OCT (Cirrus HD-OCT; Carl Zeiss Meditec AG, Dublin, CA) at approximately 4-month intervals for measurement of ONH surface topography and RNFL thickness, respectively. Significant ONH surface depression and RNFL thinning were defined with reference to Topographic Change Analysis (TCA) with HRT and Guided Progression Analysis (GPA) with Cirrus HD-OCT, respectively. Multivariate cox proportional hazards models were used to investigate whether CH is a risk factor for ONH surface depression and RNFL progression after adjusting potential confounding factors. All patients with glaucoma were followed for an average of 6.76 years (range, 4.56–7.61 years). Sixty-five glaucomatous eyes (44.5%) of 49 patients showed ONH surface depression, 55 eyes (37.7%) of 43 patients had progressive RNFL thinning and 20 eyes (13.7%) of 17 patients had visual field progression. In the cox proportional hazards model, after adjusting baseline diastolic IOP, CCT, age, baseline disc area and baseline MD, baseline CH was significantly associated with ONH surface depression and visual field progression (HR = 0.71, *P* = 0.014 and HR = 0.54, *P* = 0.018, respectively), but not with RNFL thinning (HR = 1.03, *P* = 0.836). For each 1-mmHg decrease in baseline CH, the hazards for ONH surface depression increase by 29%, and the hazards for visual field progression increase by 46%. The CH measurements were significantly associated with risk of glaucoma progression. Eyes with a lower CH were significantly associated with an increased risk of ONH surface depression and visual field progression in glaucoma patients.

## Introduction

Glaucoma is chronic, progressive optic neuropathy with progressive nature. The structural change usually precedes the functional change during the course of glaucoma. Assessment of glaucoma progression, consideration with risk factor for structural change can help clinicians customize aggressiveness of therapy to increase the chances that patients will avoid functional impairment during their lifetime^1^. Identifiable optic never head (ONH) surface depression detected with HRT was shown to generally occur prior to identifiable RNFL thinning with OCT and visual field progression in previous study^2^. Nevertheless, a significant proportion of glaucomatous eyes showed ONH surface depression alone (23.3%) or progressive RNFL thinning alone (15.8%)^2^. It is unclear if the risk factors for development of ONH surface depression and progressive RNFL thinning are different. Investigation of risk factors for glaucoma progression has been largely based on visual field assessment and elevated IOP measurement has been shown in major clinical trials to be the most important risk factor for visual field progression in glaucoma patients^3–7^. In recent studies, corneal hysteresis (CH) has also been related to visual field progression in glaucoma. CH is a measure of viscoelastic behavior of the cornea which can be measured clinically by ocular response analyzer (ORA, Reichert Instruments, Depew NY)^8^. CH is an assessment of the cornea's ability to absorb and dissipate energy. If the structural relationship between cornea and connective tissue in the posterior optic nerve head and lamina cribrosa exist, an association between CH and the functional behavior of the lamina cribrosa is conceivable, which may provide the basis that CH is related to glaucoma progression. Previous studies have shown that an eye with lower CH was associated with glaucoma progression^9–11^. In a retrospective study, Congdon et al.^9^ showed that lower CH values, but not CCT, was associated with visual field progression. De Moraes et al. reported an association between a lower CH and a faster visual field change^10^. In a recent study by Medeiros et al.^11^, they showed that baseline CH was related to the rate of change of visual field index. It remains unknown, however, if CH is also a risk factor for progressive ONH surface depression and RNFL thinning. We hypothesized that the CH was related to ONH surface depression and RNLF thinning in addition to visual field progression. The purpose of this study was to investigate if CH measured at the baseline visit predicted progressive ONH surface depression and RNFL thinning in glaucoma patients.

## Results

**Figure 1:**
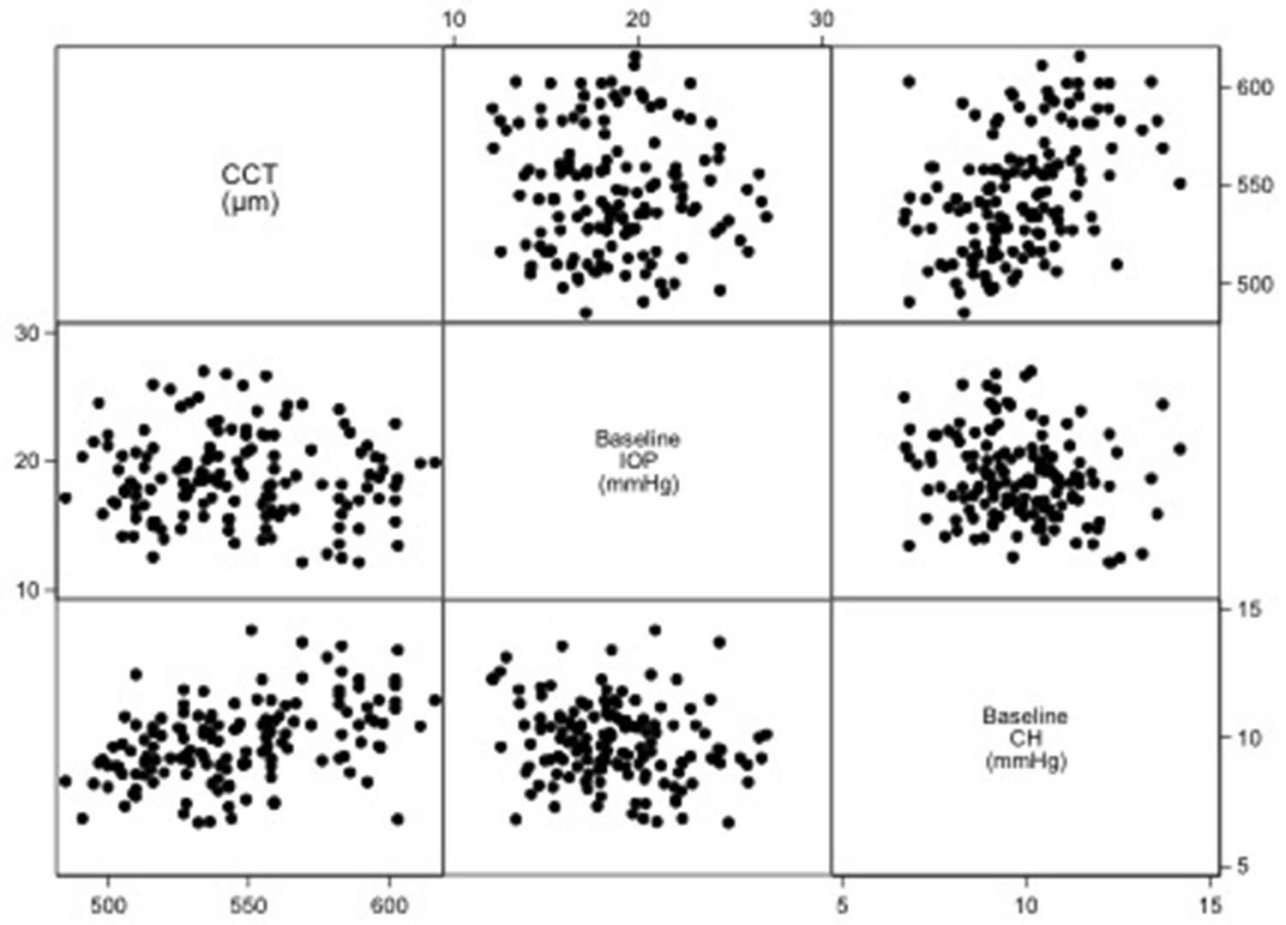
Scatter matrix illustrating the relationship among baseline corneal hysteresis (CH), central corneal thickness (CCT), and baseline dynamic contour tonometry (DCT) measurements (DCT-IOP).

The study included 146 eyes of 90 patients with glaucoma followed for an average of 6.76 ± 0.65 years (range, 4.56–7.61 years). The baseline DCT-IOP was 18.8 ± 3.3 mmHg (range, 12.1–27.0 mmHg) and baseline CH was 9.8 ± 1.5 mmHg (range, 6.7–14.2 mmHg). At the baseline examination, there were 68.7% mild (MD ≥ -6 dB), 22.5% moderate (-6 dB > MD > -12 dB) and 8.8% advanced (MD ≤ -12 dB) glaucomatous eyes. The demographics and baseline visual field, ONH, RNFL, and CH measurements of glaucoma patients are presented in Table 1. Figure 1 showing a scatter matrix illustration of the relationshi*p* among baseline DCT-IOP, CCT and CH. A significant relationship was found between DCT-IOP and CH (r = -0.19, *P* = 0.018), and CCT and CH (r = 0.43, *P* < 0.001). In this study, no significant relationship was found between age and CH (r = 0.016, *P* = 0.129).

**Table 1:** The demographics and baseline visual field, optic nerve head (ONH), retinal nerve fiber layer (RNFL), and corneal hysteresis (CH) measurements of glaucoma patients (Mean ± SD).

Parameters	
Patients/eyes	90/146
Age (years)	54.05 ± 15.11
Gender, female (%)	35 (38.9%)
Refraction (D)	− 2.93 ± 4.03
Axial length (mm)	24.78 ± 2.07
CCT (µm)	546.53 ± 30.70
Baseline DCT-IOP (mmHg)	18.76 ± 3.34
Baseline CH (mmHg)	9.84 ± 1.53
Baseline RNFL (µm)	75.88 ± 13.77
Baseline MD (dB)	− 4.90 ± 5.00
Baseline disc area (mm^2^)	2.22 ± 0.53

SD—standard deviation; D—diopter; RNFL—retinal nerve fiber layer; IOP—diastolic intraocular pressure; CH—corneal hysteresis; MD—mean deviation; CCT—central corneal thickness; dB—decibel.

Sixty-five glaucomatous eyes (44.5%) of 49 patients showed ONH surface depression, 55 eyes (37.7%) of 43 patients had progressive RNFL thinning and 20 eyes (13.7%) of 17 patients had visual field progression. Eyes with ONH surface depression had thinner CCT (540.5 ± 31.55 µm), younger age (45.05 ± 15.06 years) and smaller baseline CH (9.44 ± 1.36 mmHg) than those without ONH surface depression (551.41 ± 29.29 µm, 50.47 ± 14.33 years, 10.15 ± 1.59 mmHg, respectively) (Table 2). No significant differences in axial length, baseline DCT-IOP, baseline RNFL thickness, MD and disc area (*P* ≥ 0.174) were found between eyes with and without ONH surface depression (Table 2). Eyes detected with RNFL progression had a higher baseline diastolic IOP than eyes without (19.49 ± 3.35 mmHg verse 18.31 ± 3.27 mmHg, *P* = 0.035) (Table 3) whereas eyes detected with visual field progression had a smaller baseline CH (9.15 ± 1.59 mmHg and 9.95 ± 1.49 mmHg, respectively, *P* = 0.027) and eyes with visual field progression had relative younger ages than those without visual field progression (47.90 ± 17.32 years and 48.10 ± 14.51 years, respectively, *P* > 0.05), although the comparison was not significantly different (Table 4).

**Table 2:** Comparisons of baseline ocular biometry, visual field, optic nerve head (ONH), retinal nerve fiber layer (RNFL), corneal hysteresis (CH) and intraocular pressure (IOP) measurements (DCT-IOP) between eyes with and without optic nerve head (ONH) surface depression with CSLO.

	With ONH surface depression (65 eyes)	Without ONH surface depression (81 eyes)	P*
Age (years)	45.08 ± 15.06	50.47 ± 14.33	0.027
Axial length (mm)	25.08 ± 1.75	24.54 ± 2.8	0.174
CCT (µm)	540.45 ± 31.55	551.41 ± 29.29	0.029
Baseline (DCT-IOP) (mmHg)	18.38 ± 3.26	19.05 ± 3.39	0.224
Baseline CH (mmHg)	9.44 ± 1.36	10.15 ± 1.59	0.028
Baseline RNFL thickness (µm)	75.86 ± 12.79	77.31 ± 14.24	0.555
Baseline MD (dB)	− 4.74 ± 5.24	− 5.03 ± 4.82	0.514
Baseline disc area (mm^2^)	2.04 ± 0.42	2.02 ± 0.43	0.929

CCT—central corneal thickness; DCT-IOP—diastolic intraocular pressure; MD—mean deviation; CH—corneal hysteresis.

*Comparisons were performed using Linear mixed modeling.

**Table 3:** Comparisons of baseline ocular biometry, visual field, optic nerve head (ONH), retinal nerve fiber layer (RNFL), corneal hysteresis (CH) and intraocular pressure (IOP) measurements (DCT-IOP) between eyes with and without RNFL progression with Cirrus HD-OCT.

	With RNFL progression (55 eyes)	Without RNFL progression (91 eyes)	P*
Age (years)	49.33 ± 16.49	47.31 ± 13.82	1.000
Axial length (mm)	24.71 ± 2.02	24.82 ± 2.12	0.860
CCT (µm)	550.71 ± 31.22	544.00 ± 30.28	0.197
Baseline DCT-IOP (mmHg)	19.49 ± 3.35	18.31 ± 3.27	0.035
Baseline CH (mmHg)	9.92 ± 1.48	9.79 ± 1.56	0.587
Baseline RNFL thickness (µm)	78.40 ± 12.74	75.62 ± 14.04	0.366
Baseline MD (dB)	− 4.60 ± 4.18	− 5.08 ± 5.45	0.514
Baseline disc area (mm^2^)	2.03 ± 0.43	2.03 ± 0.42	0.794

CCT—central corneal thickness; DCT-IOP—diastolic intraocular pressure; MD—mean deviation; CH—corneal hysteresis.

*Comparisons were performed using Linear mixed modeling.

**Table 4:** Comparisons of baseline ocular biometry, visual field, optic nerve head (ONH), retinal nerve fiber layer (RNFL), corneal hysteresis (CH) and intraocular pressure (IOP) measurements (DCT-IOP) between eyes with and without visual field progression.

	With visual field progression (20 eyes)	Without visual field progression (126 eyes)	P*
Age (years)	47.90 ± 17.32	48.10 ± 14.51	1.000
Axial length (mm)	25.03 ± 2.04	24.74 ± 2.09	0.059
CCT (µm)	551.15 ± 32.39	545.79 ± 30.50	0.466
Baseline DCT-IOP (mmHg)	19.63 ± 3.70	18.62 ± 3.27	0.616
Baseline CH (mmHg)	9.15 ± 1.59	9.95 ± 1.49	0.027
Baseline RNFL thickness (µm)	77.15 ± 10.37	76.59 ± 14.06	0.617
Baseline MD (dB)	− 5.32 ± 3.57	− 4.83 ± 5.20	0.652
Baseline disc area (mm^2^)	2.02 ± 0.44	2.03 ± 0.43	0.953

CCT—central corneal thickness; DCT-IOP—diastolic intraocular pressure; MD—mean deviation; CH—corneal hysteresis.

*Comparisons were performed using Linear mixed modeling.

In the cox proportional hazards model, after adjusting baseline DCT-IOP, CCT, age, baseline disc area and baseline MD, baseline CH was significantly associated with ONH surface depression and visual field progression (HR = 0.71, *P* = 0.014 and HR = 0.54, *P* = 0.018, respectively) (Table 5). For each 1-mmHg decrease in baseline CH, the hazards for ONH surface depression increase by 29%, and the hazards for visual field progression increase by 46%.

**Table 5:** Cox proportional hazards models showing multivariate hazard ratios for optic nerve head (ONH) surface depression, retinal nerve fiber layer (RNFL) progression and visual field progression after adjusting ages, CCT, baseline DCT-IOP, average DCT-IOP during follow-up, baseline disc area and baseline MD.

	ONH surface depression		RNFL progression		Visual field progression	
	Hazard Ratio	*p*	Hazard Ratio	*p*	Hazard Ratio	*p*
Age (years)	0.98	0.067	1.01	0.364	1.00	0.993
CCT (µm)	0.99	0.270	1.01	0.407	1.02	0.058
Baseline DCT-IOP (mmHg)	0.88	0.104	1.07	0.331	1.00	0.967
Average of DCT-IOP during follow-up (mmHg)	1.10	0.275	1.06	0.474	1.03	0.808
Baseline CH (mmHg)	0.71	0.014	1.03	0.836	0.54	0.018
Baseline disc area (mm^2^)	1.56	0.265	1.12	0.770	0.68	0.605
Baseline MD (dB)	1.05	0.175	1.01	0.851	1.02	0.719

CCT—central corneal thickness; DCT-IOP—diastolic intraocular pressure; MD—mean deviation; CH—corneal hysteresis.

## Discussion

In this prospective study, we demonstrated that CH was significantly associated with ONH surface depression and visual field progression, but not with RNFL thinning in glaucoma patients. Eyes with a lower baseline CH were associated with a significantly higher risk of progressive ONH surface depression detected with CSLO and visual field progression detected with standard automated perimetry. Our finding showed that the ocular biomechanical properties are related to glaucoma progression and that measurement of corneal hysteresis is useful for risk assessment in glaucoma patients.

A number of studies have reported the association between CH and glaucomatous optic nerve head damage^12–14^. Prata et al. showed that a lower CH value was associated with larger and deeper cup in untreated glaucoma patients^14^ and correlated with greater pressure-related rim and cup volume/area change^15^. It has been suggested that optic nerve head and peripapillary sclera is more susceptible to IOP damage in eyes with a lower CH^12,14,16^. The association between baseline CH and visual field progression in glaucoma patients has also been shown by Medeiros et al.^11^. To our knowledge, this is the first study reporting the significant association between CH and ONH surface depression in glaucoma.

We showed that eyes with ONH surface depression (65 eyes) had smaller baseline CH (9.44 ± 1.36 mmHg) than those without ONH surface depression (10.15 ± 1.59 mmHg). Corneal Hysteresis is the difference in the inward and outward pressure values obtained during the patented dynamic bi-directional applanation process utilized by the Ocular Response Analyzer. It is a characterization of the cornea’s ability to absorb and dissipate energy, which is a function of visco-elastic biomechanical properties of the cornea. Low CH represents low damping ability of cornea, assuming that there is an association in the ocular biomechanical properties between the cornea and the ONH and/or lamina cribrosa (LC) surfaces, we expected eyes with a lower CH would be associated with decreasing capacity to dampen force applied to the optic nerve head. And a decreased ability to dampen a force applied to the optic nerve would certainly be consistent with increased ONH surface depression. Optic nerve head consists of optic nerve, lamina cribrosa, vessels and connective tissue and so on, the biomechanical properties of ONH and/or lamina cribrosa (LC) surfaces may not exactly the same as cornea. Further investigation is necessary to study the relationship of biomechanical properties between corneal and optic nerve head, and also glaucoma progression.

It is worth noting that CH measurement can be affected by other biometric variables, like CCT, age and IOP^17,18^. In our study, baseline CH was significantly associated with baseline IOP and CCT (*P* ≤ 0.018). We therefore included these parameters in the cox proportional hazards model. For each mmHg decrease in baseline CH, the hazards for ONH surface depression increased by 29% after adjustment of baseline diastolic IOP, CCT, age, baseline disc area and baseline MD. However, baseline CH was not associated with progressive RNFL thinning. Of note, the lamina cribrosa and peripapillary sclera are the main load-bearing structures of the ONH^19^. Progressive changes of the peripapillary RNFL may be less directly related to the ocular biomechanical properties.

Medeiros et al.^11^ performed a prospective study evaluating the role of CH as a risk factor for visual field progression in 114 eyes of 68 patients with glaucoma. They showed that a smaller baseline CH was significantly associated with a faster rate of visual field loss. In our study, visual field progression was determined based on the EMGT criteria and we obtained similar results. For each mmHg decrease in CH, the hazards for visual field progression increased by 46%. Our results corroborate the previous studies supporting that CH is a risk factor for visual field progression in glaucoma.

There are limitations of the study. First, the ability to detect progressive RNFL thinning, ONH surface depression and visual field progression could be related to the stage of glaucoma and the precision of the instrument. Second, there was no reference standard to validate and determine the sensitivity of RNFL and ONH changes detected by OCT and CSLO. Third, CH is a viscoelastic parameter, capturing the difference between loading and unloading deformation pathways. It is also unknown to what degree the material property of the cornea is related to that of the ONH structures. Finally, almost all patients in our study had received IOP lowering treatment. The sequence of RNFL, ONH and visual field changes in the natural course of glaucoma progression remains obscure.

In conclusion, eyes with a lower CH were significantly associated with an increased risk of ONH surface depression and visual field progression in glaucoma patients. This prospective study provides data supporting that baseline CH measurement is useful for evaluation of disease progression in glaucoma patients.

## Materials and methods

One hundred and forty-six eyes of 90 glaucoma patients were recruited with follow-up duration (mean: 6.76 ± 0.65 years; range: 4.56–7.61 years). All patients were followed at approximately 4-month intervals during the period from June 2007 to December 2014 at the University Eye Centre, the Chinese University of Hong Kong. The detailed protocols in accordance with the Declaration of Helsinki were approved by the institutional review board of The Chinese University of HongKong. All subjects were told of the purpose of the study and gave written informed consent before inclusion. At each follow-up visit, subjects underwent a full ophthalmic examination, visual field examination, RNFL imaging with OCT, and optic nerve head with CSLO. We confirm that all methods were carried out in accordance with clinical research guidelines and regulations of The Chinese University of HongKong. All included eyes had best corrected visual acuity of at least 20/40. Eyes with retinal pathology, macular disease and refractive or retinal surgery were excluded. Eyes with uncomplicated cataract or glaucoma filtration surgery could be included in this study. Patients with glaucoma were identified on the basis of the presence of visual field defects with corresponding optic disc and RNFL changes in at least 1 eye independent of the level of IOP. During the follow-up, patients were treated at the discretion of the attending ophthalmologists with reference to the target IOP. The progression detection algorithms–-Guided Progression Analysis (GPA) (Carl Zeiss Meditec) and Topographic Change Analysis (TCA) (Heidelberg Engineering) were used to detect progressive RNFL thinning and ONH surface depression respectively.

### Corneal hysteresis measurement with ocular response analyzer

Corneal hysteresis was acquired with ocular response analyzer (ORA, Reichert Inc, Depew, NY). For ORA measurement, a metered air pulse is delivered to the cornea and the cornea is flattened at two different time points (force-in applanation and force-out applanation). An electro-optical collimation detector system is used to record the light reflectivity from the cornea. The reflectivity is most intense when the cornea is completely flattened. CH is derived from the difference between the force-in and force-out applanation^17^. Three other parameters were also measured from each ORA signal profile: IOPcc, IOPg and CRF. Four ORA measurements were taken on an examined eye each time and the average value was calculated; only measurements with a waveform score larger than 5 were accepted.

### IOP measurement with dynamic contour tonometry

IOP measurement was taken by dynamic contour tonometry (DCT) (Pascal; Swiss Microtechnology AG, Port, Switzerland). The working principle of DCT has been described^20^. In brief, the matching of the contour of the tonometer and the contour of the cornea allows the IOP measured by a pressure sensor located at the tip of the tonometer. In this study, the recording duration of each measurement was about 5 s. Measurement with a quality score less than 3 (scale 1 to 5) was repeated until 2 measurements of quality score between 1 and 3 (as recommended by the manufacturer) were obtained in each eye and the average value was calculated. DCT records the diastolic IOP and ocular pulse amplitude (OPA) after measurement. only diastolic IOP (DCT-IOP) was analyzed in the study.

### Optical coherence tomography imaging

Spectral domain OCT imaging was performed with Cirrus HD-OCT (software version 5.0; Carl Zeiss Meditec). The acquisition rate of the Cirrus HD-OCT was 27,000 A-scans per second and the transverse and axial resolutions were 15 μm and 5 μm, respectively. An optic disc cube consisting 200 × 200 axial scans, 6 × 6mm^2^ centered on the optic disc was used to evaluate the optic disc parameters for every subject. Images were captured by operators with at least 1 years’ experience using the Cirrus HD-OCT (Carl Zeiss Meditec). The pupils were not routinely dilated during RNFL imaging. However, dilation with tropicamide 0.5% and phenylephrine 0.5% each was performed when the pupil size was too small for images of the required quality to be obtained. Images with poor centration, motion artifact, poor focus, or missing data were detected by the operator at the time of imaging, with rescanning performed in the same visit. Each OCT scan included in the study had signal strength ≥ 7. Saccadic eye movement was detected with the line-scanning ophthalmoscope overlaid with OCT en face during OCT imaging. Images with motion artifact were rescanned in the same visit^21,22^. Serial RNFL thickness maps were analyzed for detection of change using the Guided Progression Analysis (GPA)^23^.

The Cirrus HD-OCT Guided Progression Analysis (GPA) (Carl Zeiss Meditec AG) was used to analyze serial RNFL thickness maps (200 × 200 pixels) for detection of progressive RNFL thinning. Guided Progression Analysis automatically aligned and registered 2 baseline and the follow-up OCT images so that the same superpixel (1 superpixel = 4 × 4 pixels) locations could be analyzed for detection of change. The difference in RNFL measurement of an individual superpixel between the baseline and the follow-up RNFL thickness maps was compared with an estimate of test–retest variability of that particular superpixel (proprietary database from Carl Zeiss Meditec AG). Superpixels with an RNFL measurement difference exceeding the test–retest variability between a follow-up and the first and second baseline images would be encoded in yellow in the OCT RNFL thickness change map (50 × 50 superpixels). If the same changes were evident in an additional consecutive follow-up image, the superpixels would be encoded in red. In this study, the 2 baseline images were separated by approximately 4 months and progressive RNFL thinning was confirmed when an area of more than 20 superpixels (factory default) was encoded in red in the RNFL thickness change map for at least 2 consecutive visits. At least 3 consecutive follow-up visits showing significant RNFL thickness reduction were required to confirm progressive RNFL thinning.

### Confocal scanning laser ophthalmoscopy imaging

Optic disc imaging was performed with the HRT 3 (software version 3.0, Heidelberg Engineering). A three dimensional topographic image consisting of up to 384 × 384 × 64 pixels was constructed from multiple focal planes axially along the optic nerve head. An average of three consecutive scans was obtained and aligned to compose a single mean topography for analysis. The optic disc margin was outlined by an experienced examiner on the mean topographic image. Once the contour line was drawn, the software automatically calculated all the optic disc measurements. The reference plane is defined at 50 μm posterior to the mean retinal height between 350° and 356° along the contour line. The area above and below the reference plane is defined as rim and cup respectively. Re-scanning was performed in the same visit if motion artifacts were detected immediately after the imaging. All eyes included in the analysis had an image quality standard deviation ≤ 30 µm.

The HRT Topographic Change Analysis (TCA, Heidelberg Engineering) was used to analyze serial ONH topography images (96 × 96 superpixels; 1 superpixel = 4 × 4 pixels) for detection of ONH surface depression^24^. Individual superpixel ONH surface height measurements were compared between the baseline and each of the follow-up examinations with an F test. The pooled variability of the baseline and the follow-up examinations of a particular pixel was compared with the within variability of the baseline and the follow-up examinations (with an error probability of the F-test < 5%). If significant ONH surface depression was detected in a superpixel and confirmed with at least 2 consecutive follow-up visits, the superpixel would be encoded in red in the significance map. The saturation of the color increased with the magnitude of surface height change. Progressive ONH surface depression was defined using 3 criteria (liberal, moderate, and conservative) with reference to the area and depth of ONH surface depression adopted from the studies by Chauhan and colleagues^25,26^. The liberal criterion required a cluster of ≥ 0.5% of the disc area and a depth change of ≥ 20 mm; the moderate criterion a cluster of ≥ 1% of the disc area and a depth change of ≥ 50 mm; and the conservative criterion a cluster of ≥ 2% of the disc area and a depth change of ≥ 100 mm. In this study, the moderate criterion was adopted to ensure a fair specificity for ONH surface depression detection with HRT and RNFL thinning acquired with OCT^2^. The ONH surface depression was defined when the change was detected at least 3 of 4 consecutive follow-up examinations.

### Visual field examination

Visual field was obtained with the white-on-white SITA standard 24–2 strategy in Humphrey Field Analyzer II-i (Carl Zeiss Meditec). All visual fields included in the study had fixation losses, false positive and false negative errors less than 20%. Average visual field sensitivity was expressed in MD (mean deviation), as calculated by the perimetry software. A visual field defect was defined as having three or more significant (P < 0.05) non-edge contiguous points with at least one at the P < 0.01 level on the same side of horizontal meridian in the pattern deviation plot and confirmed with at least two consecutive examinations^21,22^.

Visual field progression was analyzed with event-based analysis using the Guided Progression Analysis (GPA, Carl Zeiss Meditec) according to EMGT criteria^27^. Progression was defined when there were ≥ 3 points that showed significant changes (greater than the test–retest variabilities) compared with two baseline examinations (separated by ~ 4 months in this study) for ≥ 2 or 3 consecutive tests (i.e. “likely progression” was noted in the GPA printout in the latest follow-up visit).

### Statistical analyses

Statistical analyses were performed using Stata version 12.0 (Stata Corp LP, College Station, TX). Differences in demographics, visual field, ONH, and RNFL measurements between the progressive and nonprogressive eyes were compared with linear mixed models after adjustment of correlation between fellow eyes. A multivariate cox proportional hazards model was used to investigate if baseline corneal hysteresis and other risk factors including baseline age, CCT, baseline MD, baseline disc area, baseline diastolic IOP, and mean IOP during follow-up were risk factors for progressive RNFL thinning, ONH surface depression and visual field progression. Adjusted HRs from the multivariate cox proportional hazards models were reported in this study. *P* < 0.05 was considered statistically significant.

## Data Availability

The data used during the current study are available from the corresponding author on reasonable request.
